# Molecular, morphological and functional properties of tunnelling nanotubes between normal and cancer urothelial cells: New insights from the *in vitro* model mimicking the situation after surgical removal of the urothelial tumor

**DOI:** 10.3389/fcell.2022.934684

**Published:** 2022-12-19

**Authors:** Nataša Resnik, Diana Baraga, Polona Glažar, Špela Jokhadar Zemljič, Jure Derganc, Kristina Sepčić, Peter Veranič, Mateja Erdani Kreft

**Affiliations:** ^1^ Institute of Cell Biology, Faculty of Medicine, University of Ljubljana, Ljubljana, Slovenia; ^2^ Institute of Biophysics, Faculty of Medicine, University of Ljubljana, Ljubljana, Slovenia; ^3^ Department of Biology, Biotechnical Faculty, University of Ljubljana, Ljubljana, Slovenia

**Keywords:** bladder cancer, cholesterol/sphingomyelin membrane domains, cocultures, cytoskeletal elements, motor proteins, optical tweezers, tunnelling nanotubes, mitochondria

## Abstract

Tunnelling nanotubes (TNTs) are membranous connections that represent a unique type of intercellular communication in different cell types. They are associated with cell physiology and cancer pathology. The possible existence of tunnelling nanotubes communication between urothelial cancer and normal cells has not yet been elucidated. Therefore, we analyzed TNTs formed by T24 cells (human invasive cancer urothelial cells) and normal porcine urothelial (NPU) cells, which serve as surrogate models for healthy human urothelial cells. Monocultures and cocultures of NPU and T24 cells were established and analyzed using live-cell imaging, optical tweezers, fluorescence microscopy, and scanning electron microscopy. TNTs of NPU cells differed significantly from tunnelling nanotubes of T24 cells in number, length, diameter, lipid composition, and elastic properties. Membrane domains enriched in cholesterol/sphingomyelin were present in tunnelling nanotubes of T24 cells but not in NPU cells. The tunnelling nanotubes in T24 cells were also easier to bend than the tunnelling nanotubes in NPU cells. The tunnelling nanotubes of both cell types were predominantly tricytoskeletal, and contained actin filaments, intermediate filaments, and microtubules, as well as the motor proteins myosin Va, dynein, and kinesin 5B. Mitochondria were transported within tunnelling nanotubes in living cells, and were colocalized with microtubules and the microtubule-associated protein dynamin 2. In cocultures, heterocellular tunnelling nanotubes were formed between NPU cells and T24 cells and *vice versa*. The presence of connexin 43 at the end of urothelial tunnelling nanotubes suggests a junctional connection and the involvement of tunnelling nanotube in signal transduction. In this study, we established a novel urothelial cancer-normal coculture model and showed cells in the minority tend to form tunnelling nanotubes with cells in the majority. The condition with cancer cells in the minority is an attractive model to mimic the situation after surgical resection with remaining cancer cells and may help to understand cancer progression and recurrence. Our results shed light on the biological activity of tunnelling nanotubes and have the potential to advance the search for anticancer drugs that target tunnelling nanotubes.

## 1 Introduction

Since their discovery in 2004, tunnelling nanotubes (TNTs) have attracted much attention as critical players in long-distance intercellular communication (Rustom et al., 2004). The wide variety of cargo transferred, ranging from molecules to organelles and pathogens, suggests that TNTs are involved in various cellular processes in health and disease (Sisakhtnezhad and Khosravi, 2015). Moreover, intercellular communication is critical for the initial stage of oncogenesis, making it a crucial factor in cancer pathobiology. Due to the involvement of TNTs in tumor growth, metastasis, and chemoresistance, TNTs are considered promising therapeutic targets (Lou et al., 2018; Matejka and Reindl, 2019; Roehlecke and Schmidt, 2020).

TNTs are tubular, membranous structures that connect at least two adjacent cells or cells a few microns apart (Rustom et al., 2004). The widespread presence of TNTs represents a phenomenon of universal communication. TNTs are formed in a variety of cancer cells in 2D and 3D cultures and cancer tissue biopsies (Lou et al., 2012; Pasquier et al., 2013; Antanavičiūtė et al., 2014; Thayanithy et al., 2014; Osswald et al., 2015; Patheja et al., 2015; Civita et al., 2019; Cordero Cervantes and Zurzolo, 2021). To date, TNTs have been studied in the cells of acute myeloid leukemia, laryngeal carcinoma, mesothelioma, glioblastoma, pancreatic adenocarcinoma, and colon, ovarian, breast, urothelial, and prostate cancers (Veranic et al., 2008; Lou et al., 2012; Pasquier et al., 2013; Ady et al., 2014; Antanavičiūtė et al., 2014; Osswald et al., 2015; Desir et al., 2018; Desir et al., 2019; Kolba et al., 2019; Kretschmer et al., 2019; Pinto et al., 2020). Studies have distinguished between homocellular TNTs found within one cell type and heterocellular TNTs found between different cell types (Pasquier et al., 2013; Dupont et al., 2018; Formicola et al., 2019; Kolba et al., 2019). TNTs vary in size, with a length of 10–300 μm and a diameter of 50–800 nm (Rustom et al., 2004; Onfelt et al., 2006; Sowinski et al., 2011). Actin filaments represent the basic cytoskeletal elements of TNTs (Rustom et al., 2004). Furthermore, TNTs can be either monocytoskeletal, containing only actin filaments, microtubules or intermediate filaments, or bicytoskeletal, containing combination of two cytoskeletal elements (Austefjord et al., 2014; Wang and Gerdes, 2015; Sáenz-de-Santa-María et al., 2017; Staufer et al., 2018; Formicola et al., 2019; Veranic et al., 2008). Only recently, the tricytoskeletal structure, containing actin filaments, intermediate filaments and microtubules in the same TNTs was revealed: first in urothelial TNTs (Resnik et al., 2019) and then in pancreatic tumor microtubes (Latario et al., 2020), which are TNT-related structures.

Actin- and microtubule-associated motor proteins dictate the uni- or bidirectional transfer of cargo in TNTs that enable the exchange of the material (Halasz et al., 2018), which may be involved in restoring the functions of damaged cells and mediating resistance to treatment and cytotoxicity (Pasquier et al., 2013; Wang and Gerdes, 2015; Lu et al., 2017; Desir et al., 2018). The key determinant of open-ended TNT is cytoplasmic connectivity, which can mediate the transfer of larger cargo. Conversely, close-ended TNTs have interposed gap junctions for the transfer of molecules smaller than 1 kDa. TNTs with both types of connectivity can exist in the same cell culture (Kolba et al., 2019; Sartori-Rupp et al., 2019). Moreover, membrane lipids are crucial TNT components, especially the membrane raft lipids cholesterol and sphingomyelin, which influence the mechano-elastic properties, growth, and number of TNTs (Pontes et al., 2008; Kabaso et al., 2011; Lokar et al., 2012; Toth et al., 2017).

More than 80% of cancers are derived from epithelial tissue, including urinary bladder cancer, which is the 10th most commonly diagnosed cancer worldwide (Sung et al., 2021). Because TNTs also connect urothelial cancer cells in mono- and cocultures, they are expected to be involved in cancer progression (Lou et al., 2012; Lu et al., 2017; D'Aloia et al., 2018). New insights into cancer-to-normal cell communication *via* TNTs could potentially result in the development of advanced drug delivery systems, the tracking of delivery systems with optical methods, and early-stage disease detection and treatment. Therefore, TNT communication between cancer cells and between cancer and normal cells is equally important.

In this study, we first examined spontaneously formed TNTs in monocultures (homocellular TNTs) of normal porcine urothelial (NPU) cells and invasive urothelial cancer T24 cells. The NPU cell model corresponded to normal human urothelial cells due to the structural and functional compatibility of porcine-derived NPU cells with normal human urothelial cells (Visnjar et al., 2012; Resnik et al., 2015; Višnjar and Kreft, 2015; Resnik et al., 2018; Predojevic et al., 2022). Next, TNTs were studied in cocultures (heterocellular TNTs) of NPU and T24 cells to simulate intercellular communication in cancer tissues. Our goal was to comprehensively characterize urothelial TNTs in terms of length, diameter, number, cargo, elastic properties, lipid composition, cytoskeletal elements, motor proteins, and to investigate the potential role of TNTs in bladder cancer pathology.

## 2 Materials and methods

### 2.1 Cell cultures

Cell cultures of normal porcine urothelial cells (NPU) were established from the urinary bladders of four individual pigs (Kreft et al., 2010a; Visnjar et al., 2012). NPU cells were grown in UroM medium consisting of: 50% MCDB153 medium (% v/v, Sigma-Aldrich, Taufkirchen, Germany), 50% Advanced-Dulbecco’s modified essential medium (% v/v, Gibco), 15 μg/ml adenine (Sigma), 5 μg/ml insulin (Sigma), 0.5 μg/ml hydrocortisone (Sigma), 0.1 mM phosphoethanolamine (Sigma), 4 mM glutamax (Gibco), 100 μg/ml PenStrep (Gibco), 2.5% FBS (Gibco). NPU cells from VI- X passages were used for the experiments in this study. Cultures of the T24 cell line derived from highly malignant urinary bladder carcinoma were purchased from ATCC and grown in medium composed of 50% A-DMEM (% v/v, Advanced DMEM, Gibco), 50% F-12 (% v/v, Gibco), 5% FBS (Gibco), 4 mM Glutamax (Gibco), 100 μg/ml PenStrep (Gibco). Cells were cultured at 37°C and 5% CO_2_.

In monocultures, four types of NPU and T24 cultures with seeding densities 5 × 10^3^, 5 × 10^4^, 1 × 10^5^ and 2 × 10^5^ cells/cm^2^ were established to test the conditions with optimal number of TNTs. In cocultures, four types were established with different seeding densities of cells, presented as ratios between NPU and T24 cells (Table 1). The cocultures were grown in UroM medium. On day 2 of growth, cultures were either imaged with a phase-contrast microscope (DM IL, Leica Microsystem and AxioImager Z.1, Zeiss) or processed for labelling.

**TABLE 1:** Four types of cocultures of NPU and T24 cells with different combinations of seeding densities.

Seeding density (cells/cm^2^)			The ratio between the number of NPU and T24 cells in the coculture
Coculture type	NPU cells	T24 cells	
1	5 × 10^3^	5 × 10^3^	1:1
2	5 × 10^4^	5 × 10^3^	10:1
3	2 × 10^5^	5 × 10^3^	40:1
4	5 × 10^3^	2 × 10^5^	1:40

### 2.2 Quantification of TNTs

Cells were grown on 18 × 18 mm coverslips in Petri dishes and imaged with a phase-contrast microscope. Three independent experiments were performed for the quantification of TNTs in living cells; 1,465 TNTs of T24 cells and 276 TNTs of NPU cells were manually counted. TNT length and TNT number were determined using the ImageJ program (NIH) and reported as mean ± SEM. TNT diameters were measured on images acquired with scanning electron microscope; 10 TNTs of T24 cells and 10 TNTs of NPU cells were measured.

### 2.3 Labelling

Cells were cultured at the seeding densities for the optimal TNT formation. T24 cell monocultures and cocultures were seeded at 5 × 10^3^ cells/cm^2^ and grown for 2 days. NPU cell monocultures and cocultures were seeded at either 1 × 10^5^ or 2 × 10^5^ cells/cm^2^; these densities did not significantly differ in terms of TNT number on the day 2 after seeding.

1) Triple labelling of cytoskeletal elements

NPU and T24 cells were labelled according to the triple labelling protocol for F-actin, α-tubulin, and cytokeratin 7 (CK7) (Resnik et al., 2019) and examined with the fluorescence microscope (AxioImager Z.1, Zeiss) using an oil immersion objective (63×/NA 1.4) and an ApoTome optical sectioning device (Zeiss, Germany). TNTs were classified as positive for individual cytoskeletal element when fluorescence was observed in at least one third of the entire length of a TNT. TNTs longer than 10 μm and extending without attachment to the substrate were included in the measurements.

2) Organelle labelling

MitoTracker Orange CMTMRos (M7510, Invitrogen) and LysoTracker Red DND-99 (L7528, Invitrogen) were used to stain mitochondria and lysosomes, respectively, according to the manufacturer’s instructions. Briefly, monocultures of NPU (1 × 10^5^ cells/cm^2^) or T24 (5 × 10^3^ cells/cm^2^) cells were incubated at 37 °C with MitoTracker (100 nM, 15 min) or LysoTracker (50 nM, 1 h). The triple labelling protocol for cytoskeletal elements was used to combine MitoTracker labelling with *α*-tubulin immunolabelling (Resnik et al., 2019). Cells were incubated with mouse monoclonal anti-giantin antibodies (1:1000, G1/133, Enzo Life Sciences) and secondary goat anti-mouse antibodies AlexaFluor 488 (1:500, A-11001, Thermo Fisher Scientific) for Golgi apparatus detection, according to the protocol described in (Kreft et al., 2010a) and co-labelled with MitoTracker. Imaging was performed with a fluorescence microscope (AxioImager Z.1, Zeiss) using an oil immersion objective (63 × /NA 1.4) and optical sections generation (ApoTome, Zeiss).

3) Immunolabelling of connexin 43, TOM20, dynamin-2 and motor proteins

Mono and cocultures were fixed with 4% formaldehyde for 10 min at 22 °C. After washing with PBS, blocking buffer was added (0.5% BSA, 0.1% saponin, 0.1% gelatin, 50 mM NH_4_Cl_2_, 0.02% NaN_3_) for 45 min. Cells were then incubated for 1 h with one primary antibody (rabbit polyclonal connexin 43 (1:200, C6219, Sigma)) or with two primary antibodies: (rabbit polyclonal anti-myosin Va (1:100, 3402, Cell Signalling Technology) and mouse monoclonal anti-kinesin (KIF 5B) (1:100, ab28060, Abcam) or rabbit polyclonal anti-myosin Va and mouse monoclonal anti-dynein (1:100, ab23905, Abcam)) or mouse monoclonal anti-TOM20 (1:100, 612,278, BD Transductions Laboratories) and rabbit polyclonal anti-dynamin 2 (1:100, ab65556, Abcam). The following combinations of secondary antibodies were used: goat anti-mouse AlexaFluor 555 (1:500, A21422) and goat anti-rabbit AlexaFluor 488 (1:500, A11008), and goat anti-mouse AlexaFluor 488 (1:500, A28175) and goat anti-rabbit AlexaFluor 555 (1:500, A27039), all from ThermoFisher Scientific. For labelling individual motor proteins and actin filaments, phalloidin FITC (P5282, Sigma, working solution 16.7 μg/ml) was applied in the dark for 1 h at 22 °C. The triple labelling protocol was used for double immunolabelling of α-tubulin with single motor protein or with TOM20 (Resnik et al., 2019). Cells were embedded in mounting medium with DAPI (Vector Laboratories, Peterborough, United Kingdom). Images were acquired with the fluorescence phase-contrast microscope (AxioImager Z.1). Quantification of motor protein content in the TNTs of NPU and T24 cells was performed by calculating the number of dotted fluorescence signals of each motor protein per μm of TNT, considering the straight segment of TNT without initiation and the attaching TNT segment.

4) Proximity ligation assay (PLA)

To reveal TOM20 and dynamin two colocalisation in TNTs, proximity ligation assay was conducted using a Duolink II Fluorescence kit (Olink Bioscience, Sweden) according to the manufacturer’s protocol. Cells were embedded in mounting medium with DAPI and imaged with a fluorescence microscope (AxioImager Z.1) using an oil immersion objective (63 × /NA 1.4) and optical sections were generated with ApoTome.

5) Membrane labelling

NPU or T24 cells (1 × 10^6^ cells/cm^2^) were incubated with 2.5 μL of the fluorescent lipophilic membrane dyes DiO (green; V22886) and DiI (red; D3911, both from Thermo Fisher Scientific, Waltham, MA, USA) for 30 min at 37°C in the dark. Cells were then washed three times in culture medium, and DiI-DiO-labelled NPU and T24 cell mono and cocultures were seeded (Table 1). After 2 days, cells were fixed with 4% formaldehyde for 10 min at 22 °C, washed with PBS, embedded in mounting medium with DAPI (Vectashield, Vector Laboratories), and examined with a fluorescence microscope (AxioImager Z.1, Zeiss). Three independent experiments were performed, each time we counted TNTs on the entire growth surfaces of three coverslips (technical replicates).

6) Labelling of cholesterol/sphingomyelin membrane domains

A specific protein marker for sphingomyelin/cholesterol membrane domains, ostreolysin A6-mCherry (OlyA6-mCherry), was prepared as a fusion protein using the OlyA6 sequence (UniProtKB/Swiss-Prot: P83467.2) as described in (Skočaj et al., 2014). After 2 days, live cell mono and cocultures were labelled with 2 μM OlyA6-mCherry and with 5 μg/ml Hoechst (label for nuclei), both diluted in UroM medium, for 10 min at 37 °C. Cultures were gently washed three times with UroM medium and imaged with a fluorescence microscope (AxioImager Z.1) using a water immersion objective (63×/NA 1.25) at 37 °C.

### 2.4 Live-cell imaging

NPU cells grown on MaTek dish were incubated with MitoTracker (100 nM, 15 min) at 37 °C and washed with UroM medium. The cells were then transfer into a heating chamber (Lab-Tek) at 37 °C, 5% CO_2_, and humidity on the stage of confocal microscope (LSM900, Zeiss). Imaging was performed with ZEN software and 20×/0.8 objective was used for time lapse acquisition. Images were captured every minute for 1 h, using DefiniteFocus system (Zeiss).

### 2.5 Optical tweezers

The mechano-elastic properties of TNTs were studied with laser tweezers (Tweez 250si, Aresis, Ljubljana, Slovenia) mounted on an inverted microscope (Eclipse Ti, Nikon) with a piezo stage (Nano-LPS-200, Mad City Labs, United States). The optical tweezers were set at constant optimal power so that no cell damage or bleb formation was observed (the laser wavelength was 1064 nm). The laser beam was focused into a sample chamber through a water immersion objective (60×/NA 1.00, Nikon). A sample heater was mounted onto the microscope and objective to maintain a constant temperature (37 °C).

NPU or T24 cells were grown in a polydimethylsiloxane chamber with a glass slide bottom 2 days before the experiments. Living cells in the polydimethylsiloxane chamber were placed on the microscope, and streptavidin-coated silica beads with a diameter of 1.5 μm (Kisker Biotech, Germany) were added to the cells. The beads were functionalized with the mouse monoclonal anti-integrin beta 1 antibody (1:500, ab28100, Abcam). A bead was trapped with optical tweezers and positioned on a tube for 30 s to adhere. Then the bead was retracted from the tube at the constant speed of 1 μm/s perpendicular to the tube by moving the piezo stage. The position of the bead was recorded with a digital camera (uEye, IDS Imaging, Germany) and tracked by TweezPal software (Osterman, 2010).

### 2.6 Scanning electron microscopy

T24 and NPU monocultures were fixed with 2% glutaraldehyde and 2% formaldehyde in 0.2 M cacodylate buffer and incubated for 1.5 h at 4 °C. After fixation, cells were washed overnight at 4 °C with cacodylate buffer and post-fixed for 3 h at 4 °C with 1% OsO_4_ (Serva Electrophoresis). The next day, samples were dehydrated, incubated with hexamethyldisilazane (Sigma-Aldrich), allowed to dry overnight, and spattered with gold. Cells were observed with a scanning electron microscope (JSM-840A, Jeol, Japan).

### 2.7 Image analysis and statistics

The number of TNTs in cells was analyzed using Microsoft Office Excel, Anova, and the Tukey-Kramer test. For non-homogeneous variances, Welch’s and Games-Howell’s tests were used. Statistical significance was *p* < 0.05. In all cases, at least three independent experiments were performed. NPU cell cultures were prepared from the urinary bladders of four pigs. Values are expressed as mean ± SEM. Other data were analyzed with Student’s two-tailed *t*-test (Microsoft Excel, WA). The frequency of bead attachments, tether formation, and TNT bending was analyzed using Fisher’s exact test (GraphPad Prism 5.0).

## 3 Results

### 3.1 Normal and cancer urothelial TNTs differ in dimensions

**FIGURE 1:**
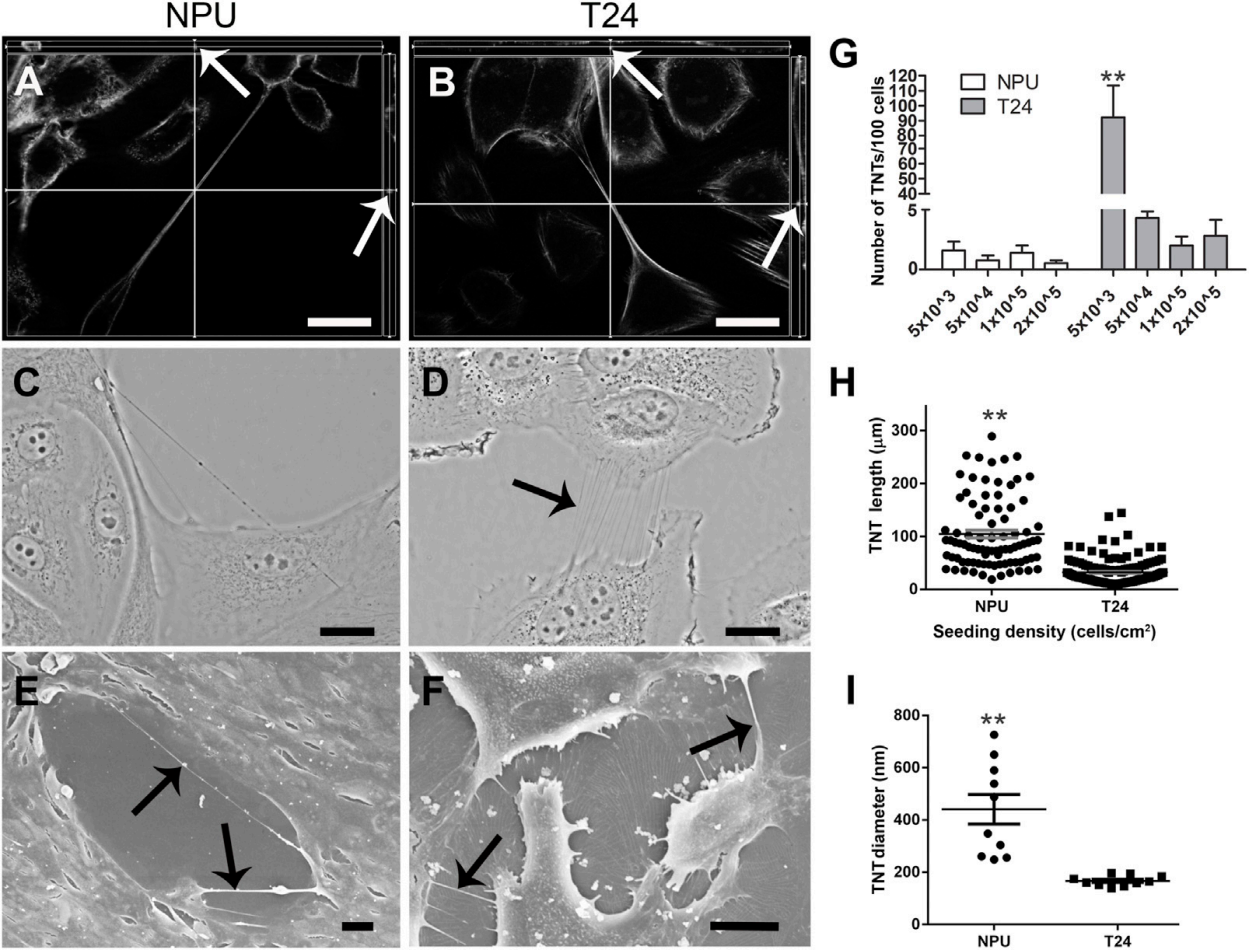
TNTs of normal (NPU) and cancer (T24) urothelial cells are morphologically different. TNTs in monocultures of NPU **(A)** and T24 **(B)** cells under fluorescence microscope **(C and D)** phase-contrast microscope, and under **(E and F)** scanning electron microscope. TNTs labelled with falloidin-FITC (gray), hover freely in the culture of NPU **(A)** and T24 **(B)** cells shown on orthoviews of optical sections (white arrows). Scale bars: 20 μm **(G)** The number of TNTs/100 cells at four seeding densities of both cell types. Numerus of TNTs counted inT24 cells was 1,456, and numerous of TNTs counted in NPU cells was 276, all in three independent experiments. **(H)** TNT lengths were measured from images of live cells acquired with a phase-contrast microscope. **(I)** The diameter of TNTs (n = 10 for each cell type) was measured from images acquired with a scanning electron microscope. Only TNTs ≥10 μm were considered for the measurements. The values in all graphs are presented as mean ± SEM. *p < 0.05; **p < 0.001.

Urothelial TNTs that did not adhere to the surface were included in the study (Figures 1A, B). To determine the number, length and diameter of TNTs in monocultures (i.e. homocellular TNTs), normal (NPU) and cancer (T24) cells were imaged with phase-contrast microscope (Figure 1C, D) and scanning electron microscope (Figure 1E, F). Types of monocultures with four seeding densities (5 × 10^3^, 5 × 10^4^, 1 × 10^5^ and 2 × 10^5^ cells/cm^2^) were used to obtain condition with the optimal (high number) TNTs for each cell type on day 2 after seeding. Regardless of seeding densities, NPU cells formed a similar number of TNTs, namely 0.6–1.6 TNTs per 100 cells (Figure 1G). The appearance and morphology of normal cells led us to decide on 1 × 10^5^ and 2 × 10^5^ cells/cm^2^ as the optimal condition for TNT formation with the best cell viability. T24 cells formed significantly higher number of TNTs, namely 92 TNT per 100 cells when seeded at 5 × 10^3^ cells/cm^2^ than in any other condition (Figure 1G). In NPU cells, the average TNT length was 104.8 ± 7.1 μm (Figure 1H). TNTs of T24 cells had a length of 32.2 ± 1.4 μm, which was significantly shorter than the length of TNTs of NPU cells (Figure 1H). Moreover, TNTs between NPU cells were also significantly thicker than TNTs between T24 cells, 441.1 ± 197.5 nm and 167.2 ± 19.8 nm, respectively (Figure 1I). The length (Figure 1H) and diameter (Figure 1I) of the TNTs were more uniform in T24 cells than in NPU cells.

Our results revealed that cancer cells formed significantly more TNTs per 100 cells than NPU cells, in the case of using the seeding density with the most frequent formation of TNTs, and that the TNT length and diameter are the characteristics on the basis of which we can distinguish between TNTs of normal and cancer urothelial cells.

### 3.2 TNTs with all three representatives of the cytoskeletal elements predominate in both normal and cancer urothelial cells

**FIGURE 2:**
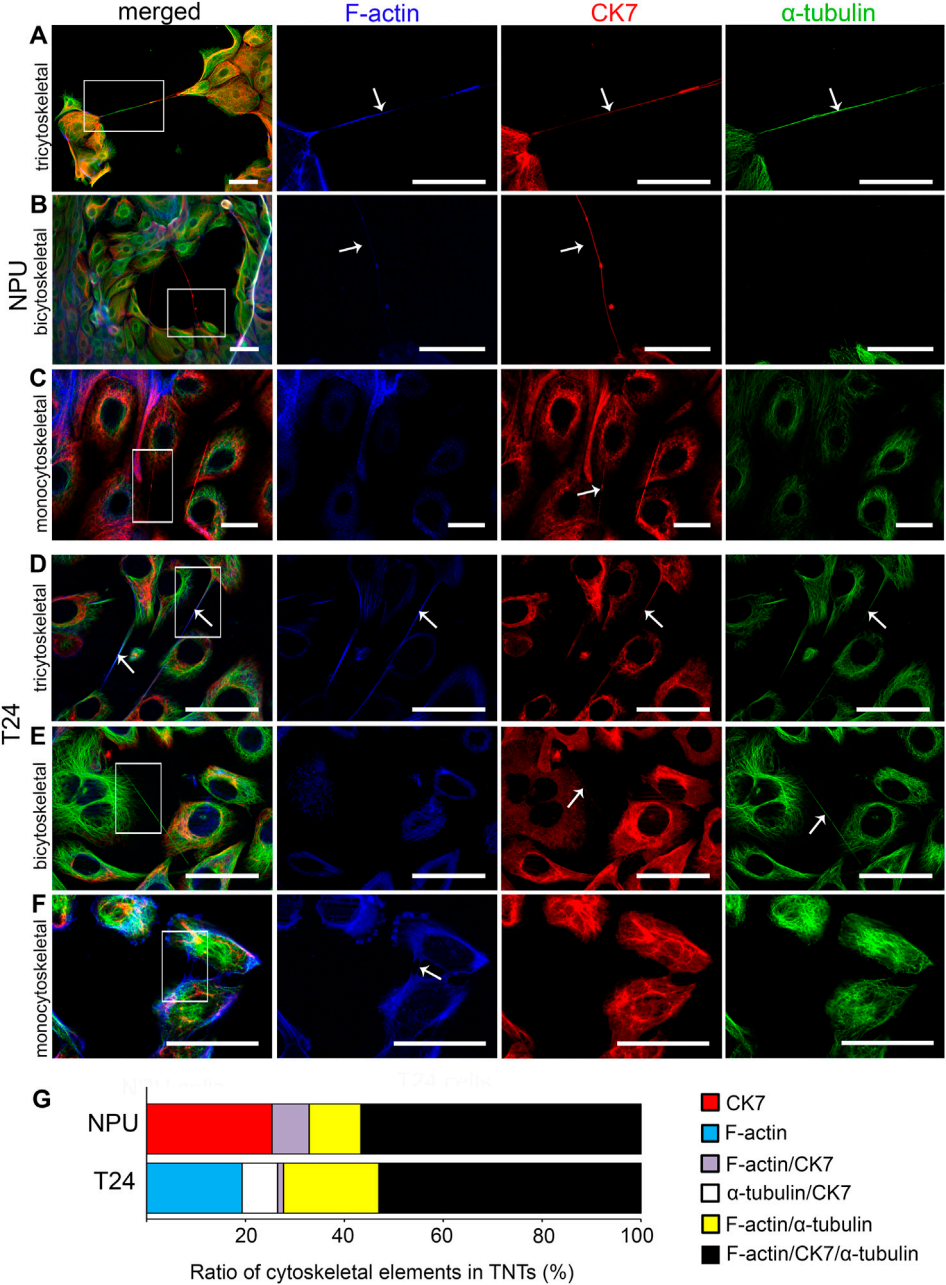
TNTs in normal (NPU) and cancer (T24) urothelial cells are mostly tricytoskeletal (**A and D**) NPU and T24 cells have three categories of TNTs regarding cytoskeletal composition: tricytoskeletal TNTs with actin filaments (blue in merged images, white in separate images), intermediate filaments (red), and microtubules (green) within one TNT (arrows), bicytoskeletal **(B and E)** and monocytoskeletal **(C and F)**. Arrows point to the cytoskeletal element that is present in the individual TNT. Scale bars: A–C 50 μm, D-E 20 μm **(G)** Proportional distributions of cytoskeletal elements in TNTs from NPU (*n* = 67) and T24 (*n* = 83) cells; percentages of different cytoskeletal combinations are presented.

In our previous study, we showed with the triple-immunolabelling that the TNTs of NPU and T24 cells contain all three cytoskeletal elements simultaneously: actin filaments, intermediate filaments, and microtubules (Resnik et al., 2019). Here, we examined the presence of F-actin (a constituent of actin filaments), CK7 (a constituent of intermediate filaments), and α-tubulin (a constituent of microtubules) (Figures 2A–F) and provided their proportional distribution (Figure 2G). In NPU and T24 cells, TNTs contained either three (Figure 2A and D), two (Figure 2B in E), or one cytoskeletal element (Figures 2C and F). Most urothelial TNTs, regardless of normal or cancerous origin, contained all three cytoskeletal elements. Specifically, in T24 and NPU cells, 53% (*n* = 83) and 57% (*n* = 67) of TNTs contained all three cytoskeletal elements, respectively, and were termed tricytoskeletal TNTs (Figure 2A and D). In NPU cells, 25% of TNTs were monocytoskeletal and contained only CK7 (Figure 2G). Bicytoskeletal TNTs in NPU cells contained CK7 and F-actin (7.5%) or α-tubulin and F-actin (10.5%). In T24 cells, the proportions of TNTs containing cytoskeletal elements were as follows: F-actin and *α*-tubulin (19%), F-actin only (19%), CK7 and α-tubulin (7%), and F-actin and CK7 (1%). In contrast to the T24 cells, the NPU cells did not contain TNTs with only F-actin, because F-actin was always present together with *α*-tubulin or CK7 or both. Conversely, T24 cells did not contain TNTs with only CK7 as NPU cells did. The immunofluorescence results showed that TNTs in T24 cells with five combinations of cytoskeletal representatives had greater variability in cytoskeletal composition than in NPU cells with four different combinations of cytoskeletal representatives.

### 3.3 Cholesterol/sphingomyelin-rich membrane domains are constituents of TNTs in cancer cells

**FIGURE 3:**
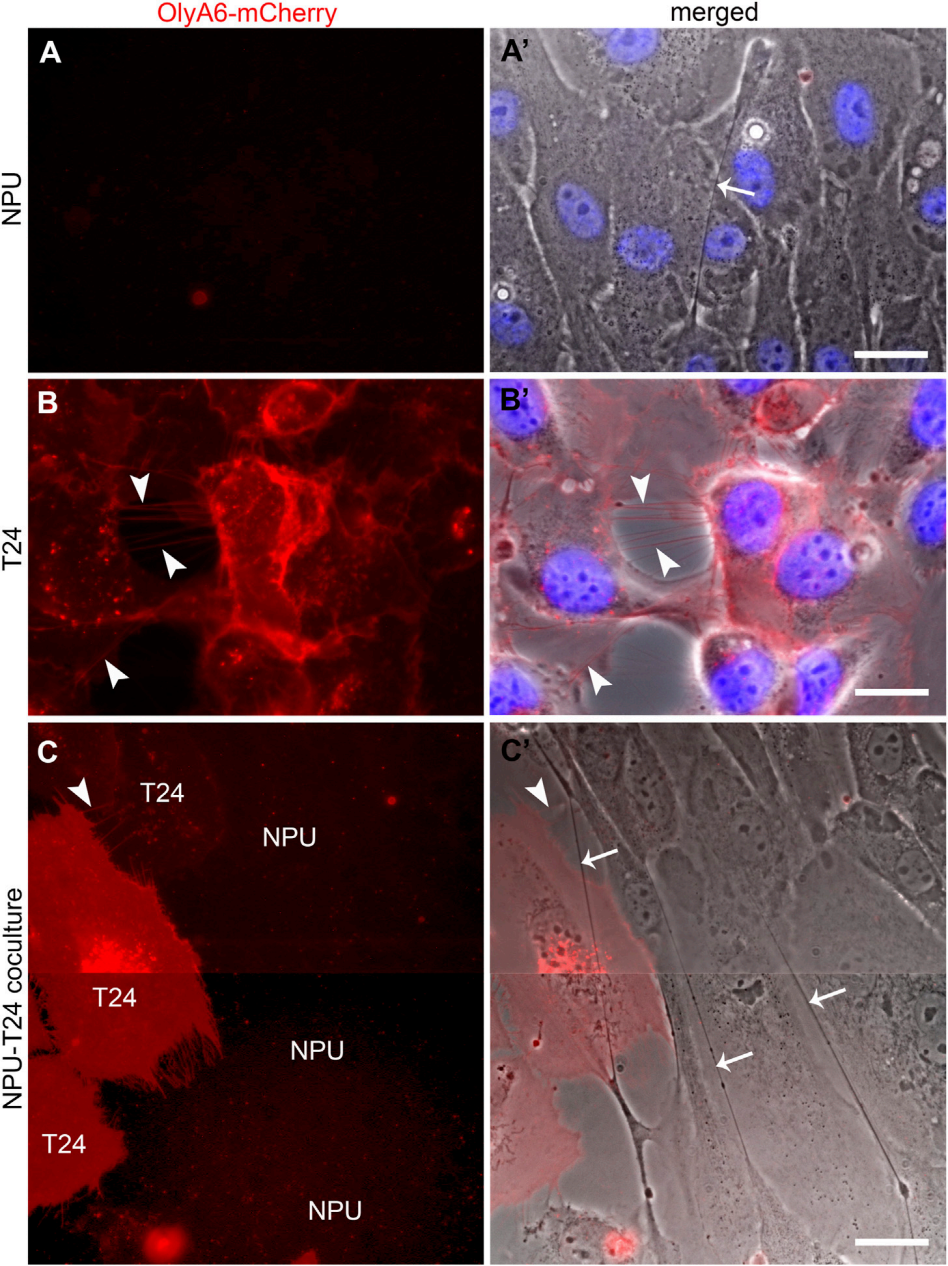
OlyA6-mCherry, a marker for cholesterol/sphingomyelin membrane domains, labels only TNTs of urothelial cancer (T24) cells. **(A)** OlyA6-mCherry does not label NPU cells in monoculture and their TNT, as shown in the merged image (**A’**, arrow). **(B)** OlyA6-mCherry labels T24 cells and TNTs (**B** and **B’**, arrowheads). Nuclei are labelled with Hoechst. **(C)** In cocultures, T24 cells are discriminated from NPU cells due to the affinity of OlyA6-mCherry to label only T24 cells and their TNTs (**C** and **C’**, arrowhead shows the array of TNTs). NPU cells and their TNTs are not labelled with OlyA6-mCherry (**C’**, arrows). Because the ascending nature of the TNTs, the cells are not perfectly in focus in the composite image. Images were acquired with a fluorescence phase-contrast fluorescence microscope. Scale bars: 20 μm.

The lipid composition of membranes dictates their mechano-elastic properties and is therefore important for the formation and stability of TNT (Delage and Zurzolo, 2013). To reveal the lipid composition of TNTs, we used OlyA6-mCherry, a protein marker that selectively binds to cholesterol/sphingomyelin membranes with cholesterol levels above ∼30 mol%, which is typical for membrane rafts (Sepcić et al., 2004; Skočaj et al., 2014). OlyA6-mCherry did not label NPU cells (Figure 3A) or their TNTs in monocultures (Figure 3A’) but did label T24 cells and their TNTs (Figure 3B, B’). In cocultures, only the T24 cells and their TNTs were labelled with OlyA6-mCherry (Figure 3C, C’).

Labelling with OlyA6-mCherry showed that the TNTs of T24 and NPU cells differed by their cholesterol/sphingomyelin membrane content. Cholesterol/sphingomyelin-enriched membrane domains were present in the TNTs of T24 cells but not NPU cells, suggesting that OlyA6-mCherry is a marker for urothelial cancer cells and their TNTs.

### 3.4 TNTs from cancer urothelial cells are easier to bend than those from normal urothelial cells

**FIGURE 4:**
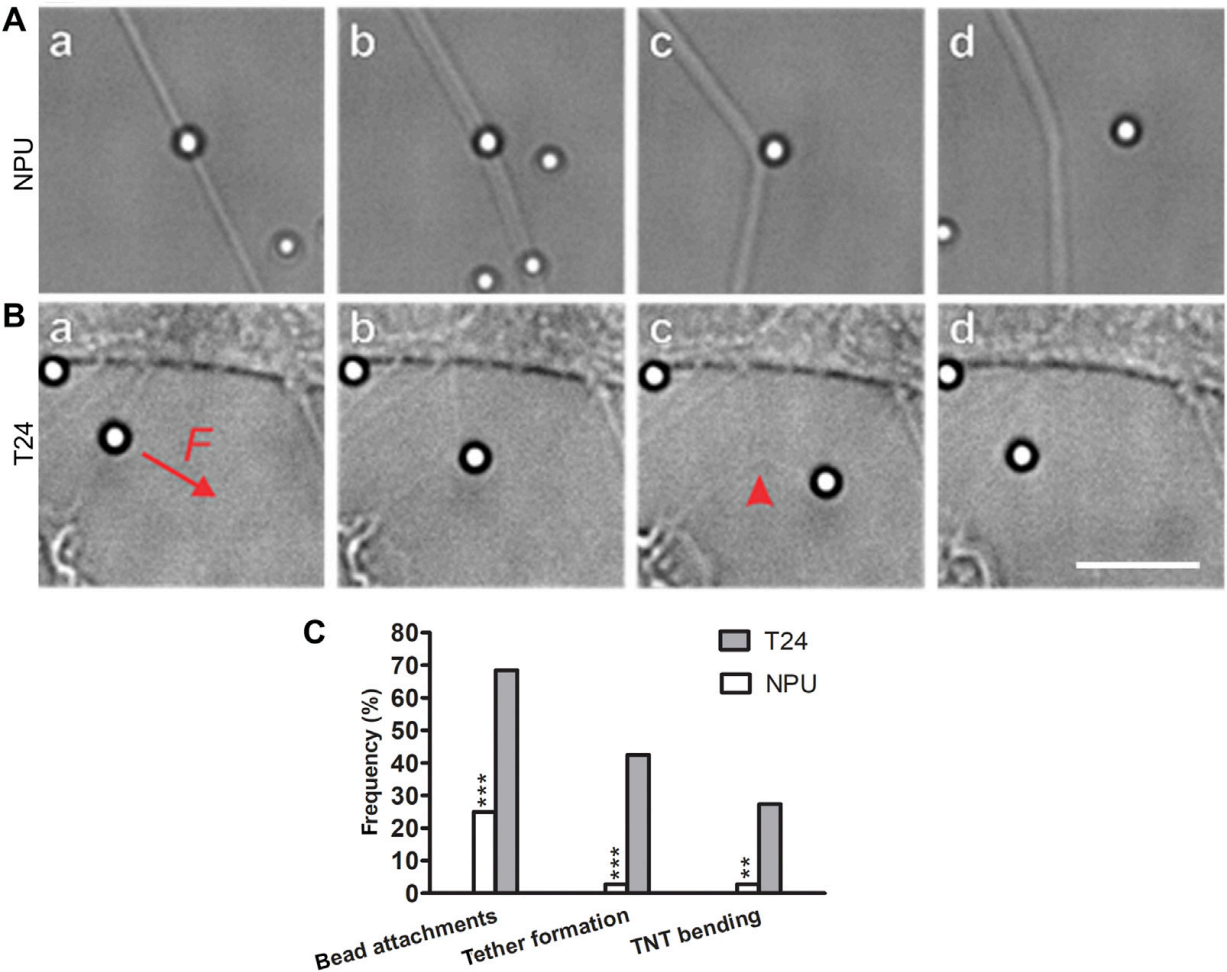
Elastic properties of TNTs in normal (NPU) and cancer (T24) urothelial cells. **(A)** An example of pulling a TNT of NPU cell (a, b, c). The TNT is less tense and remains bent even after the force subsides, when the microbead detaches (d). **(B)** A representative example of pulling a T24 cell TNT. A microbead is attached to a TNT and pulled perpendicular to the TNT (a, red arrow). The TNT bends into a tensed membrane tube with a sharp apex (b). A tether is pulled out of the TNT forming a Y-bifurcation (red arrowhead, c). After the force subsides, the TNT retracts to its initial state (d). The full sequences are available in Supplementary Videos S1, S2. Scale bar is 10 μm. **(C)** The frequencies with which the microbeads attached to the TNTs, the tether formation directly from the TNTs, and the TNT bending capability. The beads were successfully attached to 25% of NPU cells and 68% of T24 cells. The tethers were more frequently formed from the TNTs of the T24 cells (42%) than from NPU cells (3%). In addition, 3% of TNTs in NPU cells and 27.4% in T24 cells were capable of bending. F denotes force exerted by the tweezers (∼110 pN). NPU cells: n = 72 TNTs, T24 cells: n = 72 TNTs. **p < 0.01, ***p < 0.001.

The mechanical properties of TNTs from NPU and T24 cells were investigated by attaching a microbead, functionalized with anti-integrin beta1 antibody, to a TNT and pulling it perpendicular to the TNT with optical tweezers (Figures 4A, B, Supplementary Videos S1, S2). For each cell type, 72 TNTs were analyzed. Attachment of the microbeads to the TNTs was successful in 25% and 68% of cases in NPU and T24 cells, respectively (Figure 4C). Pulling on the attached microbead with the maximum possible force applied by the tweezers (∼110 pN) bent the TNTs in only two cases in NPU cells (11% of cases with successful attachment), but in 20 cases in T24 cells (41% of cases with successful attachment, Figure 4C). In addition, pulling sometimes resulted in formation of a membrane tether from TNTs, as previously observed (Pontes et al., 2008). This phenomenon did not depend on TNT bending and occurred significantly more frequently in T24 cells (42%) than in NPU cells (3%) (Figure 4C).

All bent TNTs of T24 cell exhibited a V-shape with a sharp apex at the point of the bead attachment. When the pulling ceased, the TNTs retracted back to their original shape. Thus, the TNTs of T24 cells behaved like tensed membrane tubes without any noticeable bending rigidity of the inner cytoskeletal structures (if the inner cytoskeleton provided significant mechanical rigidity against perpendicular stress, the TNTs would bend into a U-shape). Only two TNTs of the NPU cells bent during pulling, and one of them maintained the non-tensed U-shape even after the pulling force had subsided.

### 3.5 Motor proteins and organelles are present in the TNTs of normal and cancer urothelial cells: Mitochondria and microtubules are associated in urothelial TNTs

**FIGURE 5:**
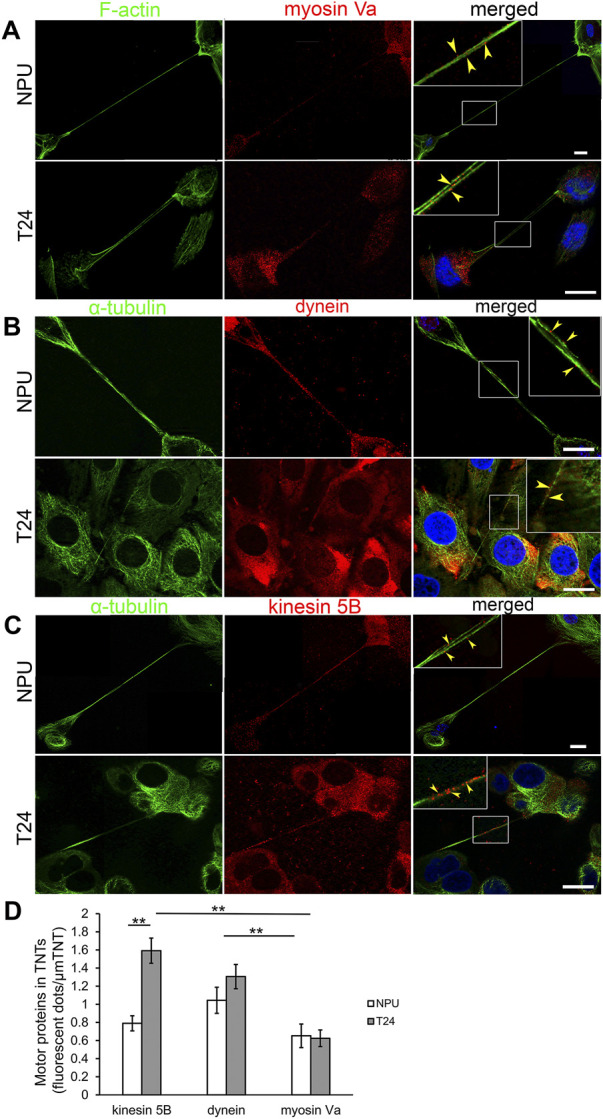
Motor proteins in TNTs of normal (NPU) and cancer (T24) urothelial cells. NPU and T24 cells contain TNTs with myosin Va **(A)**, dynein **(B)**, and kinesin 5B **(C)** along with F-actin or α-tubulin. The colocalization between motor proteins and cytoskeletal elements in TNTs (yellow arrowheads) from the insets in the merged images are magnified in the corners. The nuclei were stained with DAPI. Scale bars: 20 μm **(D)** Quantification of fluorescent dots of motor proteins (arbitrary units) per μm of TNT (mean ± SEM; NPU: kinesin, n = 17; dynein, *n* = 19; myosin, *n* = 16; T24: kinesin, n = 10; dynein, *n* = 13; myosin, n = 15; *p < 0.05, **p < 0.01).

**FIGURE 6:**
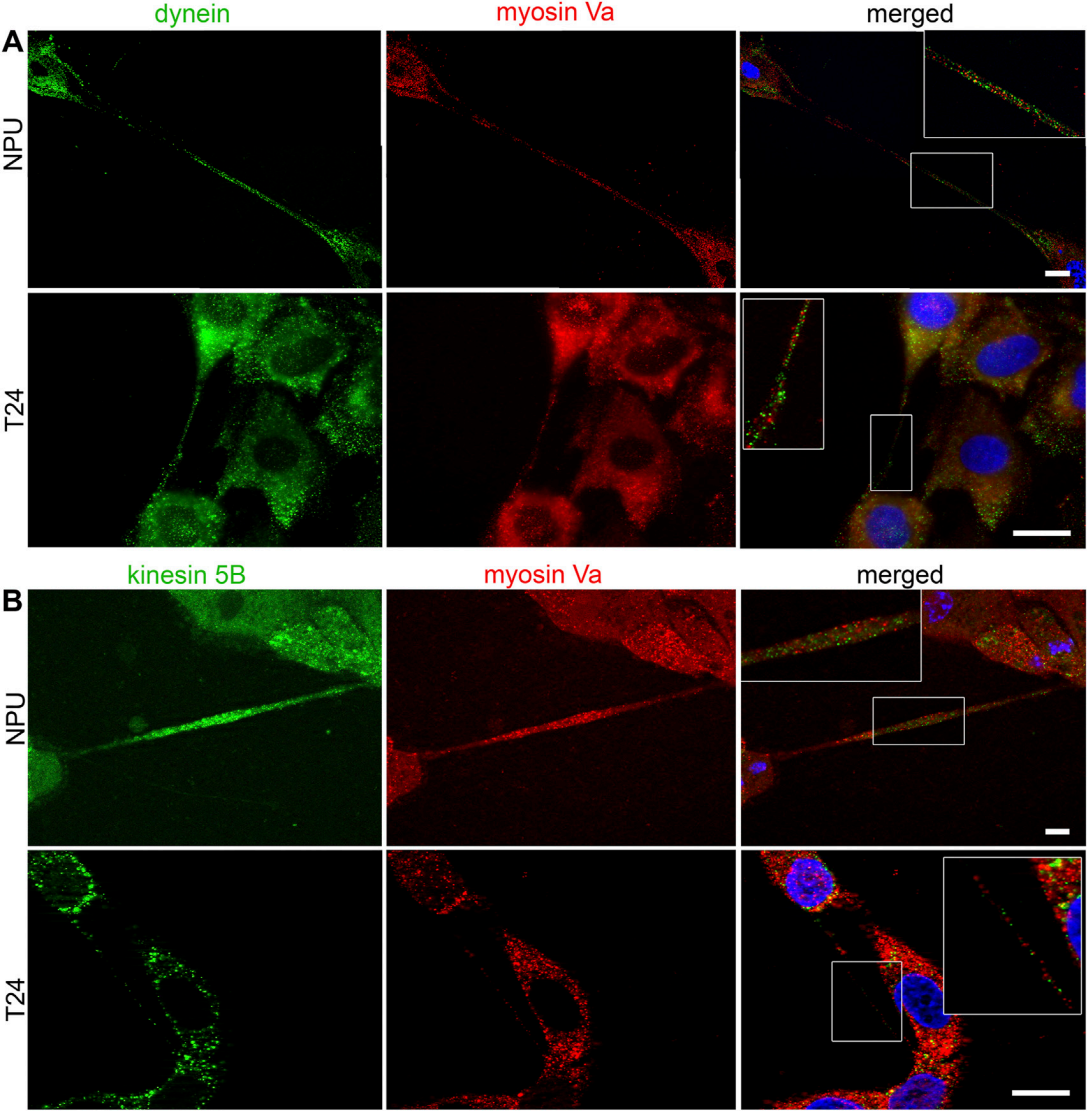
Co-distribution of motor proteins in TNTs of normal (NPU) and cancer (T24) urothelial cells. The pairs of motor proteins dynein-myosin **(A)** and kinesin-myosin **(B)** in NPU and T24 cells. The insets on the merged images are enlarged in the corners. The nuclei are stained with DAPI. Scale bars: 20 μm.

The role of TNTs in trafficking was evaluated with the immunofluorescence labelling of microtubule- (dynein and kinesin 5B) and actin-based (myosin Va) motor proteins. In NPU and T24 cells, TNTs expressed myosin Va (Figure 5A), dynein (Figure 5B), and kinesin 5B (Figure 5C), suggesting their possible involvement in trafficking. We quantified the grey values of fluorescence intensities to determine the predominant transport mechanism. Myosin Va, dynein, and kinesin 5B were equally represented in the TNTs of NPU cell (Figure 5D). In TNTs of T24 cells, the microtubule-based motor protein kinesin 5 B was more presented than in NPU cells. In T24 cells, both kinesin 5B and dynein had significantly higher fluorescence compared to actin-based myosin Va. The pairs of motor proteins dynein:myosin Va (Figure 6A) and kinesin 5B:myosin Va (Figure 6B) were present in the TNTs of NPU and T24 cells. Because dynein and kinesin 5 B drive transport in opposite directions, these results suggest that bidirectional transport occurs *via* TNTs in both monocultures.

**FIGURE 7:**
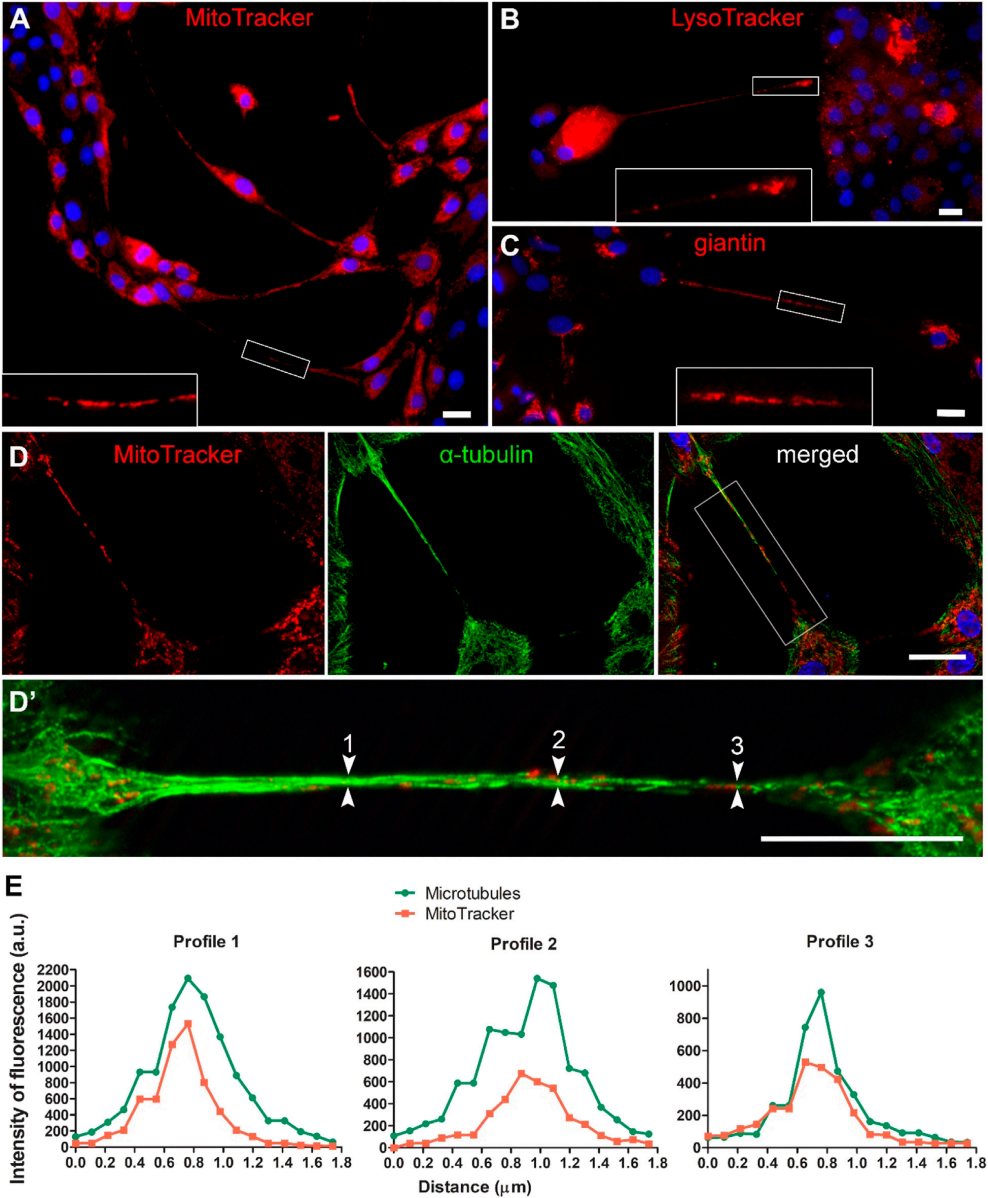
Mitochondria, lysosomes and Golgi apparatus in TNTs of normal (NPU) urothelial cells. Distribution of mitochondria (**A**,**D**,**D’**), lysosomes **(B)**, and Golgi apparatus **(C)** in TNTs. The large insets in A, B, C are 300% magnifications of the corresponding small insets. **(D)** The distribution of mitochondria and microtubules in TNTs, enlarged in (**D’**). Numbers 1, 2, and 3 (arrows) denote cross sections in TNT where profiles of fluorescence intensities of microtubules and MitoTracker were measured **(E)**. Data is presented as intensity of fluorescence (arbitrary units a. u.) per 1.8 μm cross section. Scale bars: 20 μm.

**FIGURE 8:**
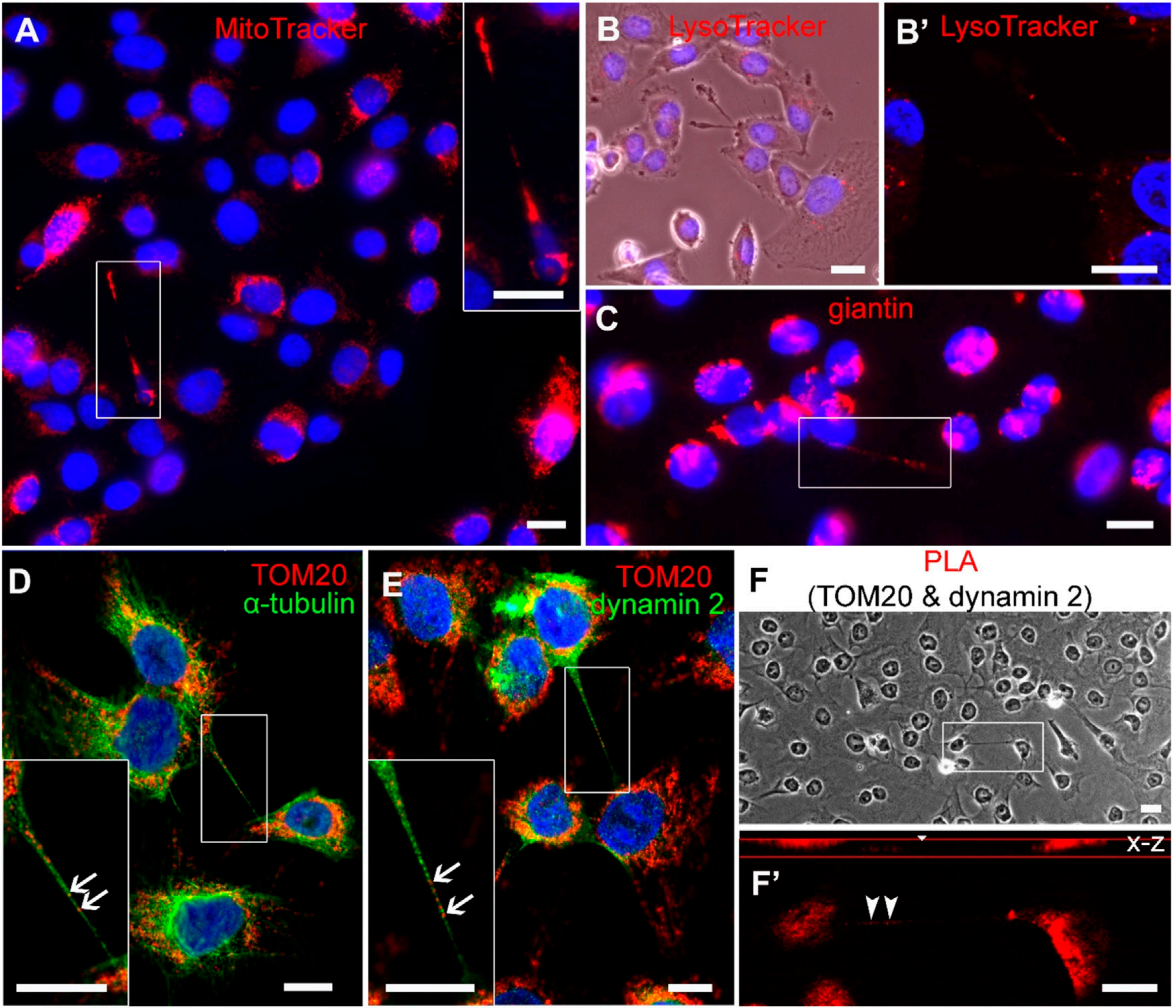
Colocalization of mitochondria and dynamin 2 in TNTs of cancer (T24) urothelial cells. Presence of **(A)** MitoTracker **(B)** LysoTracker **(C)** giantin and (**D-F’**) TOM 20 in TNTs **(D)** The distribution of TOM20 (arrows) and microtubules (green) in TNTs, and **(E)** TOM20 (arrows) and dynamin 2 (green) in TNTs. **(F)** The cells were fixed and subjected to proximity ligation assay (PLA) using anti-TOM20 and anti-dynamin 2 antibodies. **(F)** TNT in inset has positive PLA signal (**F’**, arrowheads). Scale bars: 10 μm.

Organelles are likely to be typical cargo for intercellular exchange *via* TNTs. MitoTracker (Figure 7A, D, D’, Figure 8A, D-F’), LysoTracker (Figure 7B, Figure 8B), and giantin (Figure 7C, Figure 8C) labelling revealed that the TNTs of NPU and T24 cells contain mitochondria, lysosomes, and Golgi outposts. Cross-sections of TNT at three sites (Figure 7D’) showed associated fluorescence profiles of mitochondria and microtubules (Figure 7E). In addition, TOM20, a protein belonging to the protein import machinery of the mitochondrial outer membrane, was detected in TNTs together with microtubules (Figure 8D) and microtubule-associated dynamin 2 (Figure 8E). The association between TOM20 and dynamin was investigated and proved (Figure 8F, F’) using the PLA assay, which permits *in situ* detection of protein-protein interactions at 20–100 nm proximity.

**FIGURE 9:**
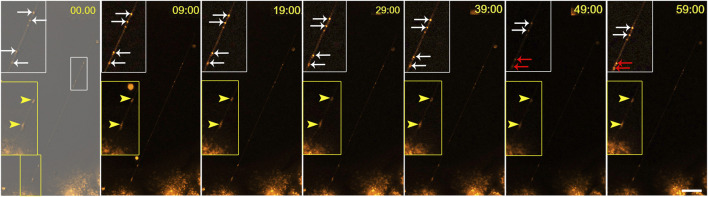
Traffic of mitochondria in TNTs. NPU cells were labelled with MitoTracker and live-cell imaging was performed for 1 h at 10-min intervals. The full sequences is available in Supplementary Videos S3. Inside the TNTs, mitochondria with different dynamics were observed: mitochondria that were moving (white arrows) and mitochondria that were not moving (yellow arrows). Red arrows denote mitochondria approaching each other. Scale bar: 20 μm.

Live-cell imaging of MitoTracker-labelled NPU cells revealed that mitochondria were transported *via* TNTs (Supplementary Video S3). Mitochondria movements are shown in sequential time-lapse images (Figure 9). The mitochondria moved in one direction during imaging at 10-min intervals for 1 h (Figure 9) and decreased the distance between each other. On the contrary, mitochondria near the end of TNT did not move during the 1-h recording (Figure 9).

### 3.6 In urothelial cocultures, porcine normal cells form TNTs to human cancer cells and *vice versa*

**FIGURE 10:**
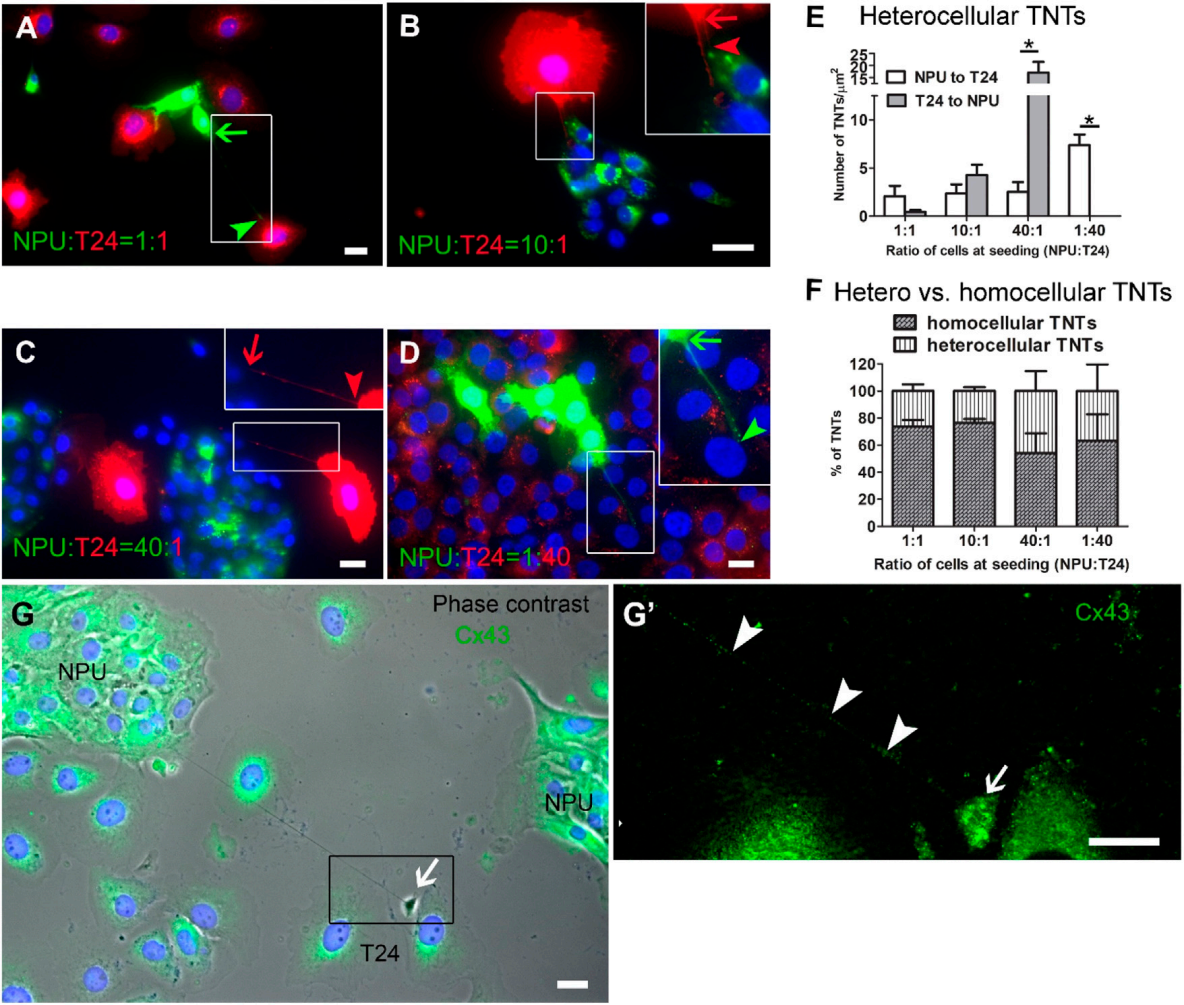
Normal urothelial (NPU) cells form TNTs to cancer urothelial (T24) cells and *vice versa* **(A)** TNTs are formed from DiO-labelled NPU cells to DiI-labelled T24 cells and from DiI-labelled T24 to DiO-labelled NPU cells. Arrows denote the beginnings and arrowheads the ends of TNTs. The color of the arrow/arrowhead denotes the cell origin of the TNT. **(A and B)** When cells were seeded at 1:1 or 10:1 ratio (NPU cells: T24 cells), the number of TNTs from NPU to T24 cells is the same as the number of TNTs from T24 to NPU cells. **(C and D)** When cells were seeded at a higher ratio (40:1 NPU:T24 cells), the number of TNTs of the cells in the minority was increased. **(C)** T24 cells in the minority formed significantly more TNTs to NPU cells in the majority **(D)** NPU cells in the minority formed significantly more TNTs to T24 cells in the majority. No mixing of dyes was detected. **(E)** The number of heterocellular TNTs/cm^2^. At seeding ratio 1:40, no TNTs from T24 cells to NPU cells were detected. **(F)** The ratio (in %) between homocellular and heterocelluar TNTs in relation to different cocultures. Ratio of cells (NPU:T24) were 1:1 (5 × 10^3^: 5 × 10^3^), 10:1 (5 × 10^4^: 1 × 10^3^), 40:1 (2 × 10^5^: 5 × 10^3^) and 1:40 (5 × 10^3^: 2 × 10^5^ cells/cm^2^). Four independent experiments were performed, in each the cells were seeded in triplicates. The entire surface with cells was imaged and all TNTs were counted. Values are expressed as mean ± SEM; *p < 0.05 . **(G)** Heterocellular TNT between NPU and T24 cell. Cx43 is present within TNT and at the end of TNT. (**G**’) The contact site is broad and enriched in Cx43. Scale bars: 20 μm.

We established cocultures of NPU and T24 cells to acquire a model of communication between cancer and normal cells. The NPU cells, labelled with membrane dye DiO, attached their TNTs to the T24 cells, labelled with DiI, and *vice versa* (Figures 10A–D), resulting in the formation of heterocellular TNTs. It is important to note that TNTs formed between cell types of porcine and human origin. Although we resuspended the NPU and T24 cells before culturing, each cell type showed a tendency to separate from the other cell type during growth, which is not uncommon in cocultures (Zani and Edelman, 2010). The quantification of heterocellular TNTs showed that the cells in the minority (those seeded at lower number), regardless of cancer or normal origin, formed more TNTs with the cells in the majority (those seeded at higher number) (Figure 10E). When ratios of NPU: T24 cells were 1:1 or 10:1, NPU cells formed TNTs with T24 cells and *vice versa*. In this condition, the number of TNTs formed from NPU to T24 cells and from T24 to NPU cells did not differ significantly (Figure 10A, B, E). When T24 cells were in the minority and NPU cells were in the majority (1:40), significantly more TNTs formed from T24 to NPU cells than from NPU to T24 cells (Figures 10C, E). When NPU cells were in the minority (1:40), they formed significantly more heterocellular TNTs to T24 cells (Figures 10D, E). In all cell ratios, the TNTs exhibited only one color and no mixing of DiI and DiO or exchange in the TNTs or cytoplasm was observed. The quantification of homocellular and heterocellular TNTs in the cocultures revealed that the ratio between homocellular and heterocellular TNTs was not statistically different in any coculture tested (Figure 10F).

These results led us to consider whether the TNT contact with the target cell is too narrow for the passage of the organelles we studied, namely membrane-labelled endosomes and mitochondria. It is known that TNTs could end up with gap junctions and allow cargo exchange below 1.2 kDa, which is below the size of organelles (Wang et al., 2010; Antanavičiūtė et al., 2014; Lock et al., 2016). Here, immunolabelling revealed the presence of gap junction protein connexin 43 (Cx43) in urothelial TNTs (Figure 10G). The enrichment of Cx43 was evident at the connection site of the heterocellular TNTs (Figure 10G’), indicating the presence of the gap junctions in TNT that terminate at the target cell.

Our results demonstrated that normal and cancer urothelial cells in cocultures form heterocellular TNTs, regardless of cell type, origin and the ratio between cells at seeding. The number of TNTs from T24 cells predominated when T24 cells were in the minority, and the number of TNTs from NPU cells prevailed when NPU cells were in the minority. The ratio between homo and heterocellular TNTs in cocultures was not statistically different. Material was transported through the TNTs, but no mixing of membrane dyes and organelles was detected, possibly due to limited cytoplasmic opening by gap junctions at the end of TNT and/or to the specific experimental setup and recording time frame.

### 3.7 Motor proteins are also found in TNTs in urothelial cocultures

**FIGURE 11:**
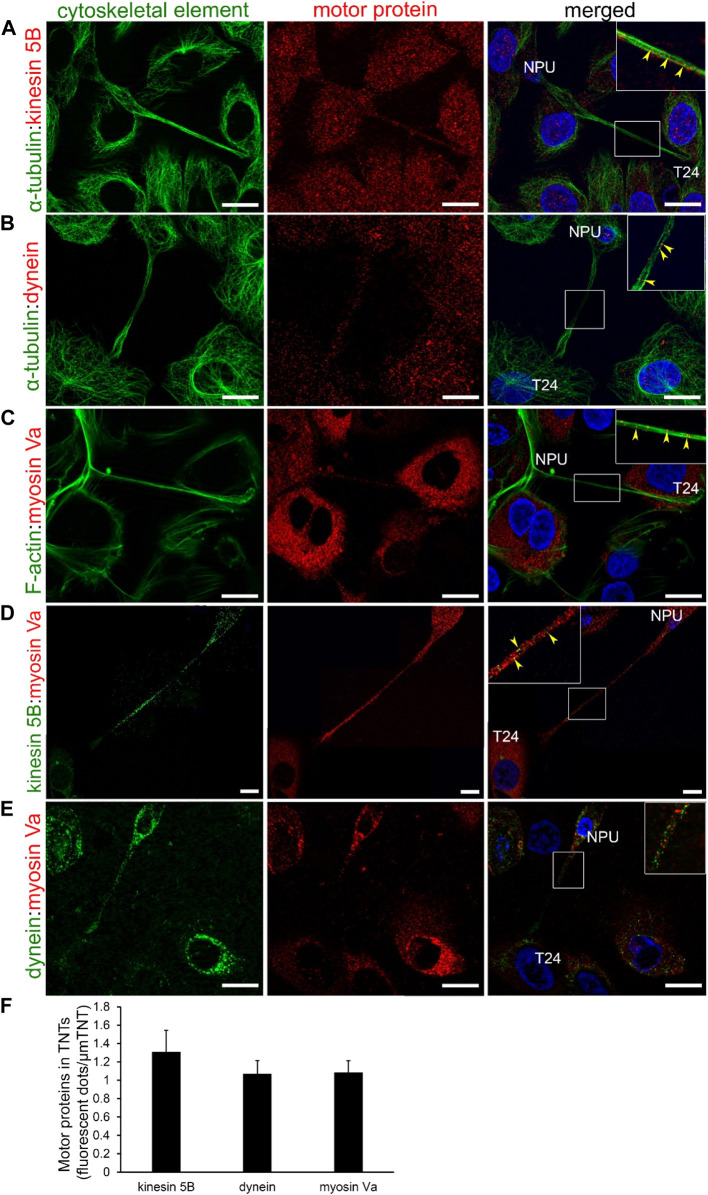
Motor proteins TNTs in cocultures of normal (NPU) and cancer (T24) urothelial cells. Motor proteins kinesin 5B **(A)**, dynein **(B)**, and myosin Va **(C)** with their associated cytoskeletal filaments were present in TNTs. Co-distribution of both motor proteins kinesin 5B and myosin Va **(D)** and dynein and myosin Va **(E)** in TNTs as detected by double immunofluorescence. The inserts on the merged images are magnified in the corners. The yellow arrowheads point to the colocalization between two motor proteins **(A–C)** or between motor proteins and cytoskeletal element **(D and E)**. NPU and T24 cells are distinguished by the size of their nuclei (stained with DAPI). Scale bars: 20 μm. **(F)** Quantification of fluorescent dots for expression of individual motor protein in heterocellular TNTs (mean of fluorescence dots/μm of TNT ± SEM; kinesin, n = 12; dynein, n = 16; myosin, n = 10).

The role of heterocellular TNTs in active transport was examined in a similar manner as of homocellular TNTs. TNTs in urothelial cocultures contained all three analyzed motor proteins: kinesin 5B (Figure 11A), dynein (Figure 11B), and myosin Va (Figure 11C). Double immunolabelling revealed the presence of actin and microtubule motor proteins within the same TNT (Figures 11D, E). Compared with monocultures, the expressions of kinesin 5B, dynein, and myosin Va were not significantly different in cocultures. Quantification of the gray values of fluorescence dots showed that the individual motor proteins, regardless of the presence of actin filaments or microtubules, were equally expressed in TNTs in cocultures (Figure 11F).

## 4 Discussion

The contribution of TNTs to cancer pathobiology is unequivocal, but still not well understood. The importance of studying heterocellular TNT interactions as crucial interface between normal and cancer cells has been highlighted (Ariazi et al., 2017). To this end, we characterized TNTs between two types of urothelial cells, normal NPU cells and invasive T24 cells in monocultures and cocultures.

First of all, NPU and T24 cells formed TNTs which significantly differed in the number, length, diameter, lipid compositions, and elastic properties. T24 cancer cells formed more TNTs than NPU normal cells when we compared the highest number of TNTs/100 cells at optimal seeding density. This trend of higher number of TNTs in cancer cells and lower number in non-cancerous cells was also found elsewhere (Ady et al., 2014; Desir et al., 2018). Then, the TNTs in T24 cells were shorter and thinner than in NPU cells and were intensely labelled with OlyA6-mCherry, a marker for cholesterol/sphingomyelin membrane domains. These domains contain >30 mol% cholesterol, which was shown in our previous studies (Sepcić et al., 2004; Skočaj et al., 2014). Interestingly, cholesterol/sphingomyelin-rich membrane domains were absent in TNTs of NPU cells. Absence of particular lipid domains in NPU cells could lie in specific protein composition of apical plasma membrane of normal urothelial cells that are in 90% covered by assemblies of uroplakins termed urothelial plaques (Wu et al., 1990; Hu et al., 2000). In cancer urothelial cells are uroplakins deficiently expressed (Kreft et al., 2010b; Zupančič et al., 2011; Višnjar et al., 2017; Kreft et al., 2022), but enriched in lipid domains (Resnik et al., 2015), giving explanation of OlyA6-negative NPU cells and their TNTs.

Although the TNTs of T24 cell were significantly shorter and thinner, they were significantly easier to bend when manipulated with optical tweezers and had more extractions of membrane tethers than TNTs of NPU cells. Several other studies have also demonstrated that the plasma membrane of aggressive cancer cells is more deformable than that of normal cells (Lekka et al., 1999; Lu and Anvari, 2020; Wang et al., 2020). Importantly, TNTs from T24 cells behaved like tensed membrane tubules, suggesting that the underlying cytoskeleton plays a negligible role in providing structural rigidity against perpendicular forces. We hypothesize that the expression of ezrin-radixin-moesin proteins, the major linkers between the plasma membrane and actin filaments in normal and cancer cells, may contribute significantly to membrane tension and tether formation (Gandy et al., 2013; Clucas and Valderrama, 2014; Rouven Brückner et al., 2015). Urothelial TNTs express cytokeratins, which confer stronger mechanical properties to TNTs than any other cytoskeletal element (Charrier and Janmey, 2016). The higher proportion of cytokeratin-positive TNTs in NPU cells than in T24 cells probably prevents TNT bending of the TNTs in NPU cells. Accordingly, pulling the TNTs of T24 cells sometimes resulted in Y-branching, which was also observed in a human glioblastoma cell line (Pontes et al., 2008). We hypothesize that bead attachment, tether formation and bending of TNTs differ between NPU and T24 cells as a consequence of the lipid composition of the plasma membrane and cytoskeleton that we uncovered in the present study, as well as the glycocalyx at the cell surface (Kuo et al., 2018), which needs further investigation.

TNT communication occurs between different cell types and different species (Lou et al., 2012; Haimovich et al., 2017; Formicola et al., 2019). In the present study, we successfully established a coculture of normal and cancer urothelial cells despite different species origin of cells (porcine *versus* human), lipid composition and mechanical properties. Our study has shown that cells that are in the minority tend to form heterocellular TNTs with cells that are in the majority and surprisingly TNT communication is not always initiated by cancer cells with more frequent TNTs. However, in cocultures, heterocellular TNTs do not predominate but are as frequent as homocellular TNTs. The condition with cancer cells in the minority is an attractive model to mimic the situation after surgical resection with remaining cancer cells and may help to understand cancer recurrence and progression. There are several recurrence mechanisms, such as undetected tumors upon cystoscopy, incomplete resection at transurethral resection of bladder tumor, tumor reimplantation after transurethral resection, and drop metastasis from upper tract urothelial carcinoma and field change cancerization (Teoh et al., 2022). Whether TNTs are involved in any of these events has not yet been proven and is limited by imaging *in vivo*.

Our quantitative analyzes of the triple cytoskeletal labelling of TNTs revealed that 57% of NPU and 52% of T24 cells contained all three cytoskeletal elements at the same time, a characteristic that has for now been described in >90% of tumor microtubes, TNT-related structures, in pancreatic cancer cells (Latario et al., 2020). We assume that the tricytoskeletal composition of TNTs is quite common feature of TNTs, but it is not routinely evidenced. Both urothelial cell types form TNTs with one, two or three cytoskeletal elements. This heterogeneity may be due to the differentiation status of the cells, the nature of TNT formation, stability and longevity. However, here we have provided the first evidence of TNTs that are positive only for cytokeratin and negative for F-actin and *α-*tubulin. Although actin filaments are indispensable constituents of TNTs, surprisingly not all urothelial TNTs were labelled with phalloidin. The reason for this could be the destruction of the actin-only TNTs during the labelling procedure, as TNTs are known to be fragile structures, especially without support from microtubules and cytokeratins. Another reason for the lack of actin labelling is its underestimation due to the low binding of phalloidin, demonstrating the limitation of the labelling procedure that could be overcome by tracking TNTs in actin-transfected living cells. It is also possible that actin is not required throughout the lifetime of TNTs with the backbone of cytokeratins and microtubules. This assumption is supported by studies in which TNTs did not break after actin-disrupting drugs (Veranic et al., 2008; Bukoreshtliev et al., 2009).

The actin- and microtubule-based transport machineries are necessary requirements for the trafficking inside TNTs. Previously, the actin-based motor proteins myosin 2, Va, VI, VIIa and X were identified in TNTs (Rustom et al., 2004; Osteikoetxea-Molnár et al., 2016; Reichert et al., 2016; Kolba et al., 2019). Here, we showed that the microtubule-based motor proteins kinesin 5 B and dynein and the actin-based motor protein myosin Va are equally represented in the TNTs of NPU cells as well as in cocultures. In contrast, in TNTs of T24 cells the microtubule-based motor proteins are more prominent than actin-based motor protein. Under steady-state conditions, we detected slow unidirectional transport of mitochondria in TNTs of living cells. The colocalization between microtubules and MitoTracker and between microtubules and TOM20, the protein of outer mitochondrial membrane, suggests microtubule-based transport machinery for mitochondrial transport. Using the proximity ligation assay, we detected colocalization in the 20–100 nm range between TOM20 and microtubule-associated dynamin 2, which is a fundamental component of the mitochondrial division machinery and a regulator of microtubule stability (Lee et al., 2016; Guo et al., 2022). The colocalization analyses revealed a strong connection/association between microtubules and mitochondria in urothelial TNTs, but further functional studies are required to confirm microtubule-driven transport.

In co-cultures, DiO-DiI labelling and OlyA6 labelling showed a distinct boundary between the donor cell TNT and associated target cells, demonstrating the absence of a membrane as well as cytoplasmic continuity. This suggests that either a) the opening is too narrow to allow organelles to pass through, b) the opening is transient, c) there is a connection mediated by gap junction leading to close-ended type of TNTs. In the present study, we found an enrichment of the gap junctional protein Cx43 in heterocellular urothelial TNTs. Cx43 plays an important role in coordinating metabolism and signaling between interconnected cells and promotes carcinogenesis in urinary bladder carcinoma (Wang et al., 2010; Abounit and Zurzolo, 2012; Poyet et al., 2015; Ariazi et al., 2017; Roehlecke and Schmidt, 2020). Recently, Cx43 has been shown to be involved in signaling pathways that govern the formation of TNTs in breast cancer cells (Tishchenko et al., 2020), and may have a similar role in urothelial TNTs. We did not detect any transfer of material between cells, probably because we only followed spontaneously occurring TNTs, where exchange normally occurs only at a low level (Yasuda et al., 2011). Similarly, studies show that cocultures of invasive and non-invasive urothelial cells (Veranic et al., 2008) and monocultures of US3 testicular cells (Jansens et al., 2017) and microvascular endothelial cells lack endosomal transfer (Latario et al., 2020). However, the opening of TNTs may become permeable to larger cellular components when close-ended TNTs transform to open-ended TNTs, which could be triggered by a chemical or physical stimulus (Gurke et al., 2008; Plotnikov et al., 2010; Yasuda et al., 2011; Astanina et al., 2015; Cervantes and Zurzolo, 2021). Whether this transformation occurs during the lifetime of urothelial TNT or is dependent on specific conditions remains to be investigated. The multilevel and controlled functionality of TNTs has been demonstrated in different cell types and is probably also present in urothelial cells.

The establishment of a coculture model of normal and cancer urothelial cells with TNT formation between both cell types provides a suitable biomimetic *in vitro* model to study pathobiology in standard 2D cultures. Our findings provide the basis for studies on 3D cultures and tissue samples. In addition, further exploration of the role of TNTs in urothelial cocultures is crucial for the potential use of TNTs as drug targets in translational medicine.

## 5 Conclusion

Our study is the first to provide a comprehensive characterization of homocellular TNTs in normal and cancer urothelial cells and heterocellular TNTs in cocultures. The major finding is the composition of three cytoskeletal elements predominant in urothelial TNTs, suggesting that all cytoskeletal elements are equally important for TNT function. Moreover, T24 and NPU cells in coculture form TNTs with each other, giving clinical relevance and potential to our study. Cancerous TNTs could be distinguished from normal TNTs by several characteristics: number, size, their increased cholesterol/sphingomyelin content, and decreased bending rigidity. Our study is a prerequisite for further exploration of TNTs between cancer and normal cells, especially in spheroids and tissue samples, with the correlation of super-resolution light microscopy and electron microscopy. Furthermore, the coculture model established in this study could explain the role of TNTs in cancer cell survival. These novel insights into the biological activity of TNTs have the potential to further advance the search for anticancer drugs that target TNTs.

## Data Availability

The original contributions presented in the study are included in the article/Supplementary Material, further inquiries can be directed to the corresponding author.
