# Supplementary material for: Molecular, morphological and functional properties of tunnelling nanotubes between normal and cancer urothelial cells: New insights from the in vitro model mimicking the situation after surgical removal of the urothelial tumor
Source: Front Cell Dev Biol. 2022 Dec 19;10:934684. doi: 10.3389/fcell.2022.934684 (PMC9806176; doi:10.3389/fcell.2022.934684)
Supplement: Supplementary file 3 [file Table1.DOCX]

**Table 1**: Four types of cocultures of NPU and T24 cells with different combinations of seeding densities.

|  | Seeding density (cells/cm^2^) | | The ratio between the number of NPU and T24 cells in the coculture |
| --- | --- | --- | --- |
| Coculture type | NPU cells | T24 cells |  |
| 1 | 5 × 10^3^ | 5 × 10^3^ | 1:1 |
| 2 | 5 × 10^4^ | 5 × 10^3^ | 10:1 |
| 3 | 2 × 10^5^ | 5 × 10^3^ | 40:1 |
| 4 | 5 × 10^3^ | 2 × 10^5^ | 1:40 |
