# Supplementary material for: Molecular, morphological and functional properties of tunnelling nanotubes between normal and cancer urothelial cells: New insights from the in vitro model mimicking the situation after surgical removal of the urothelial tumor
Source: Front Cell Dev Biol. 2022 Dec 19;10:934684. doi: 10.3389/fcell.2022.934684 (PMC9806176; doi:10.3389/fcell.2022.934684)
Supplement: Supplementary file 5 [file DataSheet1.docx]

Supplementary Material

**Tunneling nanotubes between urothelial normal and cancer cells: their molecular, morphological, and functional properties**

Nataša Resnik^1^, Diana Baraga^1^, Polona Glažar^1^, Špela Jokhadar Zemljič^2^, Jure Derganc^2^, Kristina Sepčić^3^, Peter Veranič^1^, Mateja Erdani Kreft^1^

# Supplementary Videos

Video 1: TNT pulling with optical tweezers in NPU cells.

Video 2: TNT pulling with optical tweezers in T24 cells.

Video 3: Traffic of mitochondria in TNTs. NPU cells that were grown on a glass-bottomed dish were labeled with MitoTracker (red). Cells were washed and placed in a chamber supplied with 5% CO_2_ and 37 °C with humidity on a confocal microscope (LSM900, Zeiss). Time-lapse images were recorded for 1 hour at 1-minute intervals using the software ZEN.
